# Supplementary material for: Are carbon emissions trading and green financial instruments synergistic? -Comprehensive quantitative research based on content analysis
Source: PLoS One. 2024 Mar 7;19(3):e0298601. doi: 10.1371/journal.pone.0298601 (PMC10919601; doi:10.1371/journal.pone.0298601)
Supplement: S1 Data — (ZIP) [file pone.0298601.s001.zip › Data/地级市Z_F工作报告.zip!/╔╜╢1⁄2/╤╠╠¿╩╨/└┤╫╘╣1⁄2╓┌║┼í╛┬φ┐╦╩2╛▌═°í┐_2013.docx]

各位代表：

现在，我代表市人民政府向大会报告政府工作，请予审议，并请市政协委员和其他列席会议的同志提出意见。

一、2012年工作回顾

过去的一年，是国际金融危机以来，我市经济发展极为困难的一年，也是我们应对风险挑战、推动转型升级的关键一年。全市上下在中共烟台市委的正确领导下，认真贯彻落实党的十八大精神，以科学发展为主题，以加快转变经济发展方式为主线，紧紧围绕市第十二次党代会确立的“一个率先、三个坐标、三个跨越”奋斗目标，解放思想、改革创新，积极作为、攻坚克难，经济社会发展呈现稳中有进的良好态势。

在经济发展上，产业结构调整迈出新步伐，发展质量和效益不断提高。2012年，预计全市生产总值达到5280亿元，比上年增长10.2%；完成公共财政预算收入357.4亿元，增长17.9%；服务业增加值占生产总值比重达到36.3%，提高1.4个百分点；高新技术产业产值、技术改造投资均居全省首位。在区域布局上，重大战略深入实施，“一极领先、多极崛起”发展框架初步拉开。东部海洋经济新区纳入国家海洋经济发展规划，长岛休闲度假岛发展规划获省批复，西部莱州湾、南部丁字湾、北部龙口湾开发建设整体推进。在城乡建设上，旧城旧村改造大规模展开，重大交通设施进入集中建设期。中心城市启动260万平方米旧城旧村改造工程，全市新建农房5.9万户、改造危房1.9万户。获得20亿元市级城投债发行权。潮水国际机场航站楼土建工程如期完成,青烟威荣城际铁路路基工程基本成型,龙烟铁路建设开始启动，烟海高速公路建成启用，海即跨海大桥合龙通车，15项港口工程顺利完工。胶东调水干线工程基本贯通，南水北调配套工程开工建设。在民生改善上，居民收入和保障水平进一步提高，社会保持和谐稳定。城市居民人均可支配收入突破3万元，农民人均纯收入达到13300元，分别增长13%和13.5%。12件为民服务实事全部完成。

一年来，主要做了以下工作：

（一）千方百计增强发展动力，努力保持经济增长势头。面对世界经济复苏明显放缓和国内经济下行压力加大的严峻形势，把稳增长放在更加重要位置，从扩大有效投入、挖掘消费潜力、扶持企业发展、培育经济增长点、加快功能区建设五个方面入手，增强经济增长的内生活力和动力。推动投资优化结构、稳定增长，突出抓好195个市级重点项目，全社会固定资产投资达到2970亿元，增长20.4%。增强消费能力、优化消费环境、培育消费热点，深入开展“满意消费惠万家”活动，新建改造中心城市农贸市场35处，社会消费品零售总额达到1870亿元，增长16%。努力突破企业发展制约，落实结构性减税政策，集中解决一批用地、用工、融资问题，减轻企业税费负担31.2亿元，新增中小微企业贷款190多亿元。加大财政支持经济发展力度，促进开放型经济、创新型经济、现代服务业、蓝色经济和县域经济等快速发展。实施区域发展带动战略，加快“蓝黄”两区建设，东部新区开发正式启动，龙口人工岛群围堰工程完成施工，牟平、海阳、招远等区域集中集约用海规划获得国家批复，莱阳南海新区、莱州滨海开发起步区建设顺利推进，蓬莱省级船舶制造工业园成功获批，栖霞生态经济体系建设全面展开，长岛南北长山大桥、引水济岛、电网改造工程开工建设。烟台经济技术开发区生产总值突破千亿元，烟台高新区科技创新优势不断增强，保税港区国际商品展示和保税物流功能培育成效明显，昆嵛山保护区生态建设取得新进展，招远经济技术开发区发展步伐加快。龙口、福山、莱州进入全省县域经济综合实力前十位，龙口、牟平进入省“两个同步”前十位，4个园区进入省级开发区综合评价前30强。

（二）调整优化经济结构，努力提升产业发展层次。深入实施“5510工程”和“8515工程”，海阳核电一期进入主设备安装阶段，万华工业园一期MDI工程全面开工，张裕国际葡萄酒城启动土建施工，通用东岳整车、发动机产能分别增至60万辆和120万台，华电国际莱州电厂1、2号机组并网发电，杰瑞石油、东方海洋、丛林集团、京鲁船业等一批高成长性企业快速发展，规模以上工业主营业务收入、利税、利润分别增长15%、10%和9%。新增中国驰名商标13件。中科院上海药物研究所烟台分所成功落户。引进各类人才3.3万人。获省科学技术奖43项，跻身全国首批国家知识产权示范城市。促进服务业扩大总量、提升档次,集中建设一批重点园区、特色街区、新兴项目和商贸设施。精心组织“城市月月休闲汇”活动，接待海内外游客4500万人次，旅游总收入达到460亿元，第三次荣获中国最佳休闲城市称号。成功举办和承办第六届国际葡萄酒博览会、第十三届国际果蔬•食品博览会、第六届“华交会”等130多个展会。全市港口货物吞吐量达到2.7亿吨，集装箱吞吐量达到185万标箱。开通了烟台直达韩国济州、香港等6条国际国内航线。增强农业综合生产能力和质量效益，发放涉农补贴3.8亿元，新建改造一批果园、设施蔬菜和酿酒葡萄基地，肉蛋奶、水产品产量分别达到210万吨和185万吨，粮食生产连续十年丰收。实施70座小型水库除险加固工程，解决了36.7万人饮水安全问题。

（三）加大改革开放力度，努力增强发展活力。坚持整体渐进和局部突破相结合，全面实施6大类、21项改革。国有资产监管体系更加健全，市管企业资产突破千亿元。深入实施医药卫生体制改革，龙口、蓬莱县级公立医院综合改革试点顺利推进，基本药物制度覆盖省规划的村卫生室。事业单位分类改革、经济发达镇改革、土地承包经营权和集体林权制度改革以及财政、文化体制改革取得积极进展。加快金融创新步伐，恒丰银行资产规模进一步扩大，烟台银行综合改革稳步推进，烟台农村商业银行加快筹建，引进威海商业银行设立分支机构。金融机构本外币存贷款余额分别达到5286亿元和3560亿元，增长18.2%和15.9%。中际装备、东诚生化、渤海轮渡成功上市。积极应对外需不振、外贸下滑的严峻形势，出台40多项政策措施，帮助外贸企业调结构、拓市场、稳出口、增效益，外贸进出口达到476亿美元，增长5%。组织参加香港山东周、韩国丽水世博会、台湾重点企业经贸洽谈等招商活动，与华润集团、豪德集团等大企业签约项目40多个，德尔福柴油发动机、斗山装载机研发中心等重点项目顺利实施。实现合同外资24.3亿美元，实际使用外资14.5亿美元，分别增长15%和8.5%。南山、玲珑、冰轮、烟建集团等30个企业完成境外投资2.8亿美元。新缔结3个国际友好城市。

（四）加快城乡建设步伐，努力提高城镇化水平。统筹城乡规划布局、基础设施和公共服务，基本完成新一轮城市总体规划，调整完善城镇和新农村规划。中心城市投资128亿元，实施了150项城建重点工程，胜利、滨海社区等区片改造全面展开，26个市政交通项目启动实施，11条道路竣工通车，新建了一批停车场、公交场站和过街天桥。新建改建干线公路304公里、农村公路2268公里。改造建设9处污水、垃圾处理场和1处新水厂，新增生活垃圾一体化处理村（居）578个。启动建设生态文明示范区，推行“三控两转”节能机制，单位生产总值能耗、主要污染物排放继续下降。在全国率先实现可再生能源建筑应用全域覆盖。将PM2.5纳入常规监测体系，市区环境空气质量优良率保持在90%以上。新增造林面积22.9万亩，成功创建全国绿化模范城市，再次通过全国环保模范城市复查。初步建立起土地指标分配与土地利用、综合效益、投资强度挂钩联动机制。

（五）加强社会管理创新，努力保障和改善民生。坚持经济发展与社会建设统筹推进，持续加大民生投入，全市公共财政预算民生支出增长24.6%。新增城镇就业19.5万人，农村劳动力转移就业4.5万人，荣获国家首批创业先进城市称号。企业退休人员养老待遇、居民基础养老金、城乡低保标准、新农合和城镇居民医保财政补助标准进一步提高。新增13处养老服务机构，为13万残疾人提供康复服务。素质教育不断创新，义务教育均衡发展，烟台高级师范学校、烟台二中高中部和92所乡镇幼儿园启动建设，发放困难学生资助金1.7亿元，农村中小学校车服务实现全覆盖，校舍安全工程基本完成。全市慈善义工发展到3万多人。新建和筹集保障性住房7764套。投入1.3亿元直补库区移民,建成民生工程249项。物价总水平基本稳定。“城乡共建精神文明”经验得到国家推广，在全国城市文明程度指数测评中获得好成绩。成功举办首届烟台文化艺术节，向基层免费送演出2000多场。地质博物馆建成启用，公共博物馆、图书馆、文化馆免费开放。公共体育设施加快建设，全民健身活动广泛开展。烟台运动员在伦敦奥运会取得2金、2银、打破1项世界纪录的优异成绩。第三届亚洲沙滩运动会在海阳成功举办，获得亚奥理事会、参会代表团和中外来宾的一致好评，一届精彩、简约、难忘的亚沙盛会载入亚洲体育史册，记录了烟台、海阳的奉献和光荣。坚持军民融合式发展，国防动员、民兵预备役、人民防空和双拥共建工作有序开展，烟台和长岛第七次被评为全国双拥模范城（县）。人口计生工作连续五年获得全省第一名，老龄、妇女、儿童、红十字事业健康发展，统计、史志、档案、仲裁、新闻出版、广播电视、民族宗教和气象地震等工作取得新成绩。安全生产形势保持稳定，食品药品安全状况稳中向好，应急管理水平进一步提升，有效应对了绿潮、台风、暴雪等自然灾害和突发事件。开展“社会管理创新重点推进年”活动，信访、综治、维稳工作扎实有效，社会保持和谐稳定。

一年来，我们认真执行市人大及其常委会的决议、决定，自觉接受人大、政协和社会各界监督，办理市人大代表建议和政协提案521件。积极实施“六五”普法规划，推进政务公开，深化行政审批制度改革，强化行政监察和审计监督，从严惩治腐败行为。

各位代表，过去一年，在错综复杂、快速变化的形势下，取得这样的成绩实属不易。这是党中央国务院、省委省政府和市委正确领导、科学决策的结果，是市人大、政协、各民主党派和社会各界有效监督、支持参与的结果，是全市人民团结一心、迎难而上、真抓实干的结果。在此，我代表市人民政府，向全市人民，向市人大代表、政协委员，向各民主党派、工商联、各人民团体和社会各界人士，向中央省属驻烟单位、驻烟人民解放军和武警部队官兵，向所有关心支持烟台发展的港澳同胞、台湾同胞、海外侨胞、国际友人，表示衷心的感谢，并致以崇高的敬意！

在总结成绩的同时，我们也清醒地看到，去年国内外市场环境发生重大变化，外需持续低迷，内需拉动不足，经过多方的积极努力，我市生产总值、进出口指标增幅虽都高于全省平均水平，但仍难以完成年初确定的目标；产业结构还不够优化，服务业发展提升仍不够快，自主创新能力还不够强，财政收入结构不尽合理，转方式调结构任务十分艰巨；资源要素制约日益凸现，节能减排压力加大，生态环境建设有待加强；城乡发展差距还比较大，广大市民对提高收入水平、改善生活环境、加强公共服务提出了新期待；部分干部作风不实，个别领域甚至滋生腐败现象，政府自身建设需要进一步加强。对这些问题，我们将采取有效措施，认真加以解决。

二、2013年重点工作

今年是全面贯彻落实党的十八大精神的开局之年。综观国内外形势，世界经济低速增长态势仍将延续，国内发展中不平衡、不协调、不可持续问题依然突出，但经济长期向好趋势没有改变，我国仍处于可以大有作为的重要战略机遇期。我市自然资源丰富、地理位置优越、区位优势明显，特别是几代烟台人不懈奋斗积淀下的雄厚基础，是支撑我市未来发展的最大优势。转调步伐的加快，扩大内需战略基点的确立，改革开放的深化，创新驱动发展战略的实施，是推动我市未来发展的最大动力。“蓝黄”两大国家战略的全面推进，“一极领先、多极崛起”发展格局的构建，中日韩地方经济合作示范区的设立，是促进我市未来发展的最大机遇。我们既要坚定必胜信心，又要增强忧患意识，一步一个脚印推进各方面工作，奋力开创经济社会发展新局面。

今年政府工作的总体思路是：深入学习和全面贯彻落实党的十八大精神，以邓小平理论、“三个代表”重要思想、科学发展观为指导，紧紧围绕主题主线，以“一个率先、三个坐标、三个跨越”为目标，以提高经济增长质量和效益为中心，促进工业化、信息化、城镇化、农业现代化同步发展，更加注重扩内需、稳增长，更加注重转方式、调结构，更加注重抓改革、拓开放，更加注重统城乡、保生态，更加注重惠民生、促和谐，稳中求进，开拓创新，扎实工作，努力实现经济持续健康发展和社会和谐稳定。

今年经济社会发展的主要预期目标是：全市生产总值增长10%；公共财政预算收入增长13%；全社会固定资产投资增长20%；社会消费品零售总额增长15%；实际使用外资增长8%，外贸进出口增长5%；城市居民人均可支配收入和农民人均纯收入均增长11%；城镇登记失业率控制在3.5%以内；居民消费价格涨幅控制在3%左右；人口自然增长率控制在2‰以内；完成省下达的节能减排约束性目标。

（一）牢牢把握扩大内需这一战略基点，着力增强经济发展的持久动力。坚持以改善民生为根本，以城镇化为引擎，以消费为基础，以投资为支撑，建立扩大内需长效机制。

发挥城镇化对扩大内需的拉动作用。把城镇化作为扩大内需的最大潜力，聚集资源要素、带动产业发展，完善基础设施、释放投资潜力，扩大公共服务、拓展需求空间。大力推进产业结构、就业方式、人居环境、社会保障等由乡到城的重要转变，加快建立集约、智能、绿色、低碳的城镇发展模式。实行流动人口居住证制度，积极稳妥推进户籍制度改革。

发挥消费对扩大内需的基础作用。调整收入分配制度，提高居民收入水平和消费能力。发展文化、休闲、旅游、健身等服务消费，培育一批拉动力强的消费新增长点。抓好“菜篮子”、“放心粮油”等工程，规范和降低农产品市场收费，推进红星美凯龙、招远皮革城二期、太平洋百货等一批载体设施建设，营造安全、便利、放心的消费环境。

发挥投资对扩大内需的关键作用。坚持投速、投量、投向、投效的有机统一，加大公共投资力度，引导民间投资快速增长。集中抓好200个、总投资7067亿元的市级重点项目。海阳核电1号核岛具备冷试条件，潮水国际机场完成飞行区及航站区建安工程，青烟威荣城际铁路铺轨实现预期目标，龙烟铁路开工建设控制性工程，南水北调配套工程达到进度要求。综合运用各种货币政策工具，积极拓宽融资渠道。深化银企合作，保持货币信贷合理增长，发展金融机构表外融资业务。提高城发、城投公司等平台融资能力。发挥创业投资引导作用，扶持发展融资性担保公司。支持恒丰银行优化结构、规范运营、提质增效，推动烟台银行、烟台农村商业银行深化改革、强化管理、健康发展，促进村镇银行、小额贷款公司快速兴起、扎实运作、稳步发展。认真排查和化解财政金融领域的风险隐患。金融机构贷款余额增长11%以上。

（二）突出抓好实体经济这一坚实基础，着力构建现代产业发展新体系。实施更加有利于实体经济发展的政策措施，优化生产要素配置，促进三次产业融合发展，拉长产业链条、提高附加值、培育优质品牌，推动产业层次向高端迈进。

构建更具规模、更具竞争力的先进制造业体系。继续推进“5510工程”、“8515工程”和“退城进园”工程，推动传统产业转型升级，大力培育战略性新兴产业，突出抓好100个过亿元的技改项目，加快建设万华工业园、张裕国际葡萄酒城等一批重大产业项目，推进通用东岳新型车、钛合金紧固件、航空材料产业园、绿色轮胎产业园、大金重工、海德新能源专用车等新兴产业项目，积极引进新能源汽车、云计算、3D打印、工业机器人等高端项目。推动信息化与工业化深度融合，支持发展物联网、电子商务和信息产业，努力建设智慧烟台。

构建更具活力、更具特色的现代服务业体系。按照“发展提速、比重提高、水平提升”的目标，加快发展与先进制造业配套联动的生产性服务业，培育壮大与改善民生密切相关的生活性服务业。推进总部经济基地、国际综合物流园区、服务外包示范园区、北方城大型批发市场等重点工程，建设万达城市广场、中粮大悦城等大型商贸设施，深度开发滨海海岛度假、葡萄酒休闲等十大休闲度假旅游产品，办好国际葡萄酒博览会、国际果蔬•食品博览会等展会活动，挖掘胶东鲁菜文化，叫响“鲁菜之都”品牌。力争服务业增加值占比再提高1.4个百分点以上。

构建更加安全、更加高效的现代农业体系。全面落实强农惠农富农政策，在稳定粮食生产的基础上，推动粮油、蔬菜、水果、畜牧、水产等优势产业提档升级，扶持现代种业、绿茶产业、生态药业、观光农业等特色产业发展。提升农业物质技术装备水平，创新农业经营体制机制，发展壮大农业龙头企业。加强绿色生产，新增“三品一标”认证基地15万亩、循环农业示范基地20万亩。实施10座大中型和45座小型水库除险加固工程，基本完成长岛引水济岛工程，抓好惠及29万人的农村饮水安全工程，继续改善库区生产生活条件。

构建以品牌为支撑的产业体系。培强万华、张裕、招金、泰和新材等驰名品牌，振兴传统优势品牌，扶持新兴知名品牌，提升区域优质品牌，推介农业特色品牌，引进国际著名品牌，带动提高企业、产业、城市的知名度和影响力。

构建有利于扩大财源的产业体系。扶持壮大工业主体税源，积极培育现代服务业新兴税源，深入挖掘地域特色潜力税源，完善税源控管体系，抓好企业主辅分离和资产资源性收入征管，打造财源建设新亮点，提高公共财政预算收入占生产总值比重。

（三）纵深推进改革开放创新，着力激发各类市场主体发展新活力。坚持把改革开放创新贯穿于科学发展全过程，努力推动改革实现新突破、开放迈上新台阶、创新取得新进展。

深化各领域改革。坚持顶层设计与基层首创相结合，从解决当前突出矛盾出发，研究制定重点领域改革方案，启动新一轮综合改革。完善国有资产监管体制机制，深化国有企业改革，建立国有资本经营预算制度。调整市区财政管理体制和财政分配关系。支持非公有制经济发展，推动民营企业快速膨胀，新增小微企业和个体工商户2.5万户以上。适时推进营业税改征增值税工作。加快小城镇改革试点步伐，深化农村综合配套改革。推进行政管理体制改革，稳步实施事业单位分类改革。

提升开放型经济水平。实行更加积极主动的开放战略，推动开放向优化结构、促进创新、挖掘深度、扩宽领域、提高效益的方向转变。坚持出口与进口并重，壮大十个传统出口产品集群，培育十个新兴出口产品集群，扶持重点外贸企业和出口基地健康发展，扩大先进技术装备、重要资源进口，实现对外贸易转型升级。坚持引资与引技引智并重，加强对日韩、港台、欧美、东南亚及京津沪等重点区域招商，强化与德国巴斯夫公司、韩国现代集团、台湾鸿海集团、香港招商局和港中旅等知名企业的深层次合作，推进23个投资过亿美元的重点项目，力争完成150家外资企业年度增资任务。坚持利用外资与对外投资并重，支持企业到境外建设资源性开发企业、生产加工基地和承揽工程项目。加快筹建国际航运服务中心，提高通关便利化水平。扩大文化、科技等领域对外交流与合作，推进中韩地方经济合作示范区率先起步。坚持引进外资与引进内资并重，强化面向央企和行业龙头企业的招商引资工作。

实施创新驱动发展战略。加强国家创新型城市、知识产权示范城市建设，实施科技创新“六个十工程”，发展高新技术产业，完善以企业为主体、市场为导向、产学研相结合的技术创新体系。发挥企业家、科学家才能，加强产品创新、品牌创新、管理创新、产业组织创新、融资体制创新和商业模式创新。积极引进科研院所、高等院校和跨国公司设立科研机构，支持驻烟高校、科研机构提高科研水平和成果转化能力。落实高端人才引进“双百计划”，组织美国硅谷华商博士烟台行、第七届“华交会”等活动，加快培育创新型人才和高技能人才。

（四）集中力量实施重大战略，着力培育区域发展新优势。坚持“蓝”“黄”“高”相结合，大力推进重点板块开发，形成城乡一体、海陆统筹、错位发展、有序竞争的开发局面。

加快构建“一极领先、多极崛起”发展格局。东部新区,以金山港区为重点，拉开“七纵三横”路网管网框架，启动海洋产权交易中心、海洋经济总部基地和海洋创业服务大厦建设，同步推进产业招商，在高端项目引进上取得突破。西部莱州湾，重点建设滨海开发起步区。南部丁字湾，配套完善海阳亚沙文化旅游集聚区和莱阳南海新区基础设施。北部龙口湾，加快实施龙口集中集约用海项目，全面推进招远滨海科技产业园建设。高标准建设蓬莱西海岸文化新区。有序推进长岛休闲度假岛保护性开发建设。

打造蓝色经济优势板块。大力实施“以港强市”战略，推进“港口、产业、城市”一体化布局、联动式发展，优化德龙烟铁路沿线“一线七港”功能布局，规划建设以海港、空港、保税港区和铁路中转站为依托的西部海岸临港产业区，培育壮大石油化工、临空经济、冷链物流等特色产业。推进芝罘湾港区货运功能向西港区转移，精心规划和着力打造芝罘湾高端商务区。继续关注和持续推动中韩汽车陆海联运、中韩铁路轮渡等项目。加快推进100个蓝色经济重点项目，突出发展海洋生物医药化工、海洋装备制造等现代海洋产业，支持中集来福士、大宇造船等50户海洋骨干企业做大做强，海洋产业产值增长16%以上。

推动各类园区进位升级。巩固提升烟台经济技术开发区龙头地位，在国家级开发区中领先发展；支持烟台高新区创建蓝色经济创新型特色园区，在国家级高新区中争先进位；放大保税港区政策优势，主要指标高于全国保税港区平均水平；增强昆嵛山保护区生态优势，实现文化、休闲、旅游等多产业融合发展；促进招远经济技术开发区快速崛起、跨越发展；扶持栖霞台湾农民创业园聚集资源、突破发展；推动省级园区实现五年发展新目标。

提升县域经济发展水平。支持各县市发挥优势、突出特色，发展实体经济和主导产业，走精农、强工、兴贸、富民之路。坚持“促强扶弱带中间”，促进龙口在全省县域城市中领先发展，推动莱州、招远、蓬莱、海阳赶超发展，帮扶栖霞、支持长岛加快发展，再造莱阳发展新优势。推进重点镇改革创新和整合发展，建设一批产业大镇、经济强镇、区域重镇和物流商贸、文化旅游特色名镇。

（五）不断提高城镇化质量，着力开创城乡建设新局面。以“提质加速、城乡一体、统筹发展”为目标，努力构建以中心城市为龙头、县域城市为骨干、重点城镇为支撑、农村社区为基础的新型城镇发展格局。

健全城乡规划体系。编制《烟台市城镇化发展纲要》，统筹兼顾城乡产业、用地、居住以及生态保护、基础设施和公共服务，科学规划老城区、新城区、新社区和新农村布局，精心编制重要区域和节点控制性详细规划，在全国率先建立数字化规划管理系统。

改善城乡居民居住条件。整体策划、积极推进、分步实施棚户区、工矿区、危旧建筑和农村住房改造，今年集中开展中心城市白石、慎礼、北马路等18个区片和39个村居改造工程，开工建设安置房200万平方米。新建农房6万户，改造危房8154户。完善促进房地产业健康发展的长效机制，加强保障性住房建设与分配监管，筹建各类保障性住房3200套。

打造中心城市新亮点。高标准实施芝罘、福山、莱山、牟平生态新城开发建设，加快城市科技广场、恒隆广场、华润中心等公共设施和商贸综合体建设，适时启动烟台山、朝阳街、所城历史文化街区维护性改造，积极推进大南山、养马岛、芝罘岛、崆峒岛保护性开发。引导建设一批城市景观小品，打造依山、滨海、沿河景观带，抓好特色建筑、主干道沿线灯光设计。

完善城乡基础设施。推进胜利路南延、通林路等40条城市道路建设，新建续建一批立交桥、过街天桥和停车场。优先发展公共交通，拓展和延伸公交线路及服务，将老年人公交车免费乘坐优惠范围放宽到65周岁以上。实施11项供水、供气、供热及排水、排污工程，新增供热并网面积100万平方米、燃气用户2万户，加快中石油天然气烟台段管网铺设进度。完成威青线等7条公路境内路段大修改造，开工建设潮水国际机场连接线和9条公路改造工程，规划建设6个公路与铁路换乘枢纽，新建改建农村公路400公里。

优化城乡环境。深入开展净化、绿化、亮化、美化和治脏、治乱、治堵、治污工作，提高城市精细化管理水平，改善老城区及城乡结合部基础设施和生活环境，实施市府小区、华茂小区等区片综合整治改造，抓好70余处涝洼地段改造和9条河道清淤维修工作。创新交通组织管理，营造畅通、安全、文明、便捷的交通环境。完成第二批800个村庄环境综合整治任务。

（六）全面增强文化软实力，着力构建更高水平的城市文明。坚持把文化建设与文明建设结合起来，丰富群众精神文化生活，巩固全国文明城市成果，争创国家历史文化名城。

创新发展文化事业和文化产业。深化文化体制改革，繁荣新闻出版、广播电视等文化事业，培育1861广告创意产业基地、烟台动漫基地等文化产业集聚区。加大文化投入，实施文化惠民工程，创建全国公共文化服务体系示范区。加强策划，整合资源，推出精品，积极扩大烟台特色文化影响力。精心筹办第十届中国艺术节和第二届烟台文化艺术节。

健全文明城市建设长效机制。加强社会主义核心价值体系建设，开展群众性精神文明创建活动，促进文明城市建设实效化、制度化、常态化。加强公民道德工程建设，深化道德领域突出问题专项教育和治理，发挥“道德讲堂”作用,树立先进典范，培育知荣辱、讲正气、做奉献、促和谐的良好风尚。

（七）大力推进生态文明建设，着力打造美丽烟台。牢固树立尊重自然、顺应自然、保护自然的理念，推进绿色、循环、低碳发展，建设美丽城市、美丽乡村。

加强国土资源管理。推进土地节约集约利用，控制开发强度，优化空间布局，完成单位生产总值建设用地下降目标。严格保护耕地，严肃查处违法违规用地行为。坚持集约用海，整治修复海域、海岛和海岸环境，争创全国海洋生态文明示范区。

全面促进资源节约。完善节能调控机制和能耗量交易机制，加强重点领域用能管理，继续淘汰落后产能。实行最严格的水资源管理制度，建设节水型社会。支持节能低碳产业和新能源发展，组织开展百家企业节能低碳行动，推动200家企业开展清洁生产，抓好循环经济示范园区建设，强化公共机构节能管理，增强全社会节能意识。

加大自然生态系统和环境保护力度。深入实施蓝天、碧水、青山、绿地“四大生态”工程，强化水资源、大气、土壤等污染防治，加强饮用水源地保护和重点流域综合整治，抓好水土流失和裸露山体恢复治理，完成水系造林绿化11.5万亩。按照减量化、无害化的要求，严格控制化肥农药使用，减少农业面源污染。实施一批污染物减排重点工程，更新180辆污染较重的公交车辆，实时公布PM2.5空气质量状况。

（八）在改善民生和创新管理中加强社会建设，着力办好为民服务实事。把保障和改善民生放在更加突出位置，在保基本、强基层、广覆盖、提标准、重均衡、建机制上下工夫，努力让群众过上更好生活。

实施更加积极的就业政策。完善就业服务体系，免费培训失业人员和农民工3万人以上。开发4000个公益性岗位，促进高校毕业生、城镇困难人员、退役军人等重点群体就业。善待和支持小微企业发展，强化大企业社会责任。加强创业型城市建设，扶持大学生、留学生创业园等孵化基地。新增城镇就业12万人以上，农村劳动力转移就业2.5万人以上。

统筹推进城乡社会保障体系建设。完善社会保险制度，做好社会保险关系转移接续工作，继续提高企业退休人员基本养老金。加大被征地农民社会保障力度。启动实施新农合和城镇居民大病保险制度。构建以居家为基础、社区为依托、机构为支撑的社会养老服务体系，鼓励社会力量发展养老事业和老龄产业,新增机构养老床位4000张以上。完善社会救助体系，建成5处县级社会福利中心。加强残疾人服务体系建设，实施残疾儿童“春苗阳光”康复工程，做好流浪儿童救助工作。

办好人民满意的教育。深入实施素质教育，均衡发展义务教育，大力发展学前教育，提升普通高中教育水平，优化职业教育资源，鼓励高等院校合作办学、内涵式发展。解决好农民工子女入托、入学问题。加快烟台工贸技师学院等新校区建设，建好133处乡镇公办幼儿园。强化校车管理，抓好校安工程。确保义务教育阶段学生免费校服覆盖面达到学生数的70%。继续提高师德水平和业务能力。

不断提高群众健康水平。深化医药卫生体制改革，推进县级公立医院综合改革试点，扩大基本药物制度实施范围。优化中心城市医疗资源布局，健全城乡卫生服务网络。实施体育惠民工程，推进群众体育、竞技体育、体育产业协调发展，创建全民健身示范城市。

加强和创新社会管理。抓好民主法制建设，深化社会管理综合治理，健全社会矛盾预防化解机制，增加智能化治安防控体系建设投入，严厉打击违法犯罪行为,建设和谐稳定模范城市。严格落实安全生产责任制,强化食品药品安全监管，抓好气象服务和防震、防汛、防台风及抗旱减灾等工作,有效预防和处置各类突发公共事件。大力支持国防和军队建设，搞好人民防空进社区活动。组织开展第三次经济普查。做好计划生育工作，积极发展妇女、儿童、红十字和慈善事业，推进民族宗教、史志档案等工作。

各位代表，为更好地了解民意、倾听民声、汇集民智，去年我们集中一段时间开展了“我为《政府工作报告》建言献策暨2013年‘为民服务实事’征集意见”活动，部分意见建议已纳入《政府工作报告》内容和“为民服务实事”范围，其他意见建议正在研究、解决、落实。我们将全面兑现向广大市民做出的承诺，努力使发展成果更多、更快、更公平地惠及全市人民。

三、加强政府自身建设

（一）解放思想、提升境界，增强推动科学发展能力。坚持以党的十八大精神为指引，深刻领会科学发展观的科学内涵、精神实质和根本要求，提高把握和运用市场经济规律、自然规律、社会发展规律能力，坚持用发展和改革的办法解决前进中的问题。树立全球化视野、国际化眼光、现代化标准，把各项工作放在全国、全省和首批沿海开放城市的大格局中找差距、定位置、寻坐标，瞄准先进、奋力学赶、争创一流，不断创造新的业绩。

（二）改进作风、埋头苦干，以良好的政风取信于民。严格落实中央政治局关于改进工作作风、密切联系群众的八项规定和省、市委的具体要求，从市政府领导班子做起，带头改进工作作风，带头深入基层调查研究，带头密切联系群众，带头解决实际问题。市政府领导班子成员到基层开展工作，一律轻车简从、减少陪同、简化接待。精简会议、文件，提高政务活动实效。倡导务实求实，力戒空谈浮夸。加强公务员管理，健全目标体系、考核办法、奖惩机制，严肃查处行政不作为、乱作为等问题，切实提高行政效能和服务质量。

（三）依法行政、科学决策，进一步提高行政水平。认真执行人大及其常委会的决议、决定，自觉接受人民政协的民主监督，扎实做好人大代表建议和政协提案办理工作。虚心听取各民主党派、工商联、各人民团体和人民群众的意见建议，广泛协商、广集民智，增进共识、增强合力。深入开展“行政程序年”活动，健全科学民主决策机制。转变政府职能，创新行政管理方式，提高政府执行力和公信力。深化行政审批制度改革，精简、规范行政审批事项。加大政务公开力度，高度重视网络舆情，努力改进政府工作。

（四）廉洁勤政、执政为民，营造风清气正的政务环境。严格落实党风廉政建设责任制，扎实推进惩治和预防腐败体系建设。强化廉政教育与监督，深化领导干部经济责任审计。牢固树立艰苦奋斗思想，坚持勤俭节约，严控“三公”经费支出。加强政府投资项目监管，推进公共资源交易平台建设。严肃查处违纪违法案件，坚决惩治腐败，努力做到干部清正、政府清廉、政治清明。

各位代表，今年将是风险和困难较多、机遇和挑战并存的一年。让我们紧密团结在以习近平同志为总书记的党中央周围，在中共烟台市委的坚强领导下，凝聚力量，开拓创新，勇于实践，攻坚克难，为全面建成小康社会、率先基本实现现代化，早日把烟台建设成为蓝色、文明、幸福之都而努力奋斗！
